# Targeting the gut-kidney axis to improve kidney transplantation prognosis: from mechanisms to clinical intervention strategies

**DOI:** 10.1080/0886022X.2026.2642487

**Published:** 2026-03-17

**Authors:** Huanhuan Cao, Jing Sun, Yi Lv, Jiajia Ye, Yumei Wang, Huajun Jiang

**Affiliations:** Department of Nephrology, Union Hospital, Tongji Medical College, Huazhong University of Science and Technology, Wuhan, China

**Keywords:** Kidney transplantation, gut-kidney axis, intestinal immune cells, microbiota, fecal microbiota transplantation

## Abstract

Kidney transplantation is an important treatment for end-stage renal disease, but lifelong immunosuppression is needed to prevent immune rejection, but the immunosuppressive therapy increases the risk of post-transplant complications. Therefore, how to improve the long-term survival of transplanted kidneys and reduce rejection has become a hot spot in current research. Recently, the ‘gut-kidney axis’ has received widespread attention as an important pathway for immune regulation. It refers to the fact that changes in either side of the gastrointestinal tract and kidney will affect the other side through energy metabolism, immuno-inflammation, intestinal mucosa, intestinal flora, among others, up and including to adverse consequences, which can be mutually causative. With the theory of ‘gut-kidney axis’, more and more studies have found that intestinal immune cells and microbiota play an important role in maintaining immune homeostasis and regulating the immune microenvironment of renal transplant recipients. Some studies have found that intestinal immune cells and microbiota not only influence the systemic immune status, but also may regulate the immune response of transplanted kidneys through metabolites and inflammatory mediators. In this review, we summarize the potential mechanisms of intestinal immune cells and microbiota in immune tolerance and rejection after renal transplantation based on the theory of ‘gut-kidney axis’. In addition, we highlight microbiome modulation strategies, particularly dietary interventions and fecal microbiota transplantation, as emerging approaches with potential to improve transplant outcomes. A deeper understanding of the mechanism of action of the gut-kidney axis will provide new ideas and therapeutic targets for immunomodulation after renal transplantation.

## GRAPHICAL ABSTRACT

**Figure:**
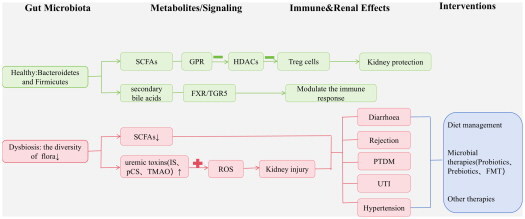

## Introduction

Kidney transplantation is an effective treatment for chronic end-stage renal disease (ESRD), which can significantly prolong the survival time and improve the quality of life of patients. In recent years, with the advancement of surgical techniques and the optimization of immunosuppressive regimens, the short-term survival rate of kidney transplantation has been significantly improved. However, long-term graft renal function maintenance still faces many challenges, including rejection, infection, drug toxicity, and immune-related complications. Among them, the maintenance of immune homeostasis is considered a key factor in achieving long-term graft survival [1].

In recent years, the interaction between the gut and the kidney has received increasing attention, and a new concept of ‘gut-kidney axis’ has been developed. The gut-kidney axis refers to a bidirectional regulatory mechanism between the intestinal microecosystem and the kidneys, which is mediated by immune and metabolic pathways, forming a systemic regulatory network that affects systemic immune and metabolic homeostasis [2]. The gut-kidney axis has been shown to be closely associated with the development of a variety of diseases, such as chronic kidney disease (CKD), hypertension, IgA nephropathy, kidney stones, and autoimmune diseases [3]. Similarly, the gut, as the largest micro-ecosystem and an important immune organ, contains a rich community of immune cells and a complex microbial ecosystem, which are also involved in regulating the health status of renal transplant patients.

Kidney transplant recipients (KTRs) are highly susceptible to intestinal flora dysbiosis and disturbances of the immune microenvironment after transplantation due to long-term immunosuppressive therapy, antibiotic exposure, lifestyle and dietary changes, and intestinal dysfunction. These alterations are associated with a range of clinical complications, including acute rejection, infections, diarrhea, and renal interstitial fibrosis [4]. Therefore, this review aims to systematically synthesize current evidence on the roles of gut microbiota and intestinal immune cells in shaping the post-transplant immune microenvironment, critically evaluate the translational potential of gut-kidney axis-targeted intervention strategies, and highlight key challenges that must be addressed to advance microbiome-based immunomodulation toward individualized and precise transplantation immunomodulation.

## Basic composition and regulatory mechanisms of the gut- kidney axis

### Composition and function of intestinal microecology

The gut of a healthy individual is inhabited by more than 100 trillion microorganisms, mainly bacteria, viruses, fungi and archaea. Bacteria are the most abundant, mainly in the colon, and are dominated by *Bacteroidetes* and *Firmicutes*, as well as *Actinobacteria* and *Proteobacteria* [5]. Intestinal flora plays an important role in maintaining the integrity of the intestinal barrier, promoting nutrient absorption, synthesizing vitamins, scavenging toxic compounds, regulating the immune system and producing metabolites, which are involved in the maintenance of host health [6]. In renal transplant recipients, a series of processes such as surgical stress, long-term use of immunosuppressants and antibiotics, and lifestyle changes often lead to structural disturbances in the intestinal flora [7,8], which are manifested by a decrease in the diversity of the flora, a decrease in the number of beneficial bacteria, the emergence of new dominant bacteria, and dysfunction of the flora, which induce consequences such as damage to the intestinal barrier, up-regulation of inflammatory factors, and immune activation [9].

### Composition and function of the intestinal immune system

**Figure 1.**
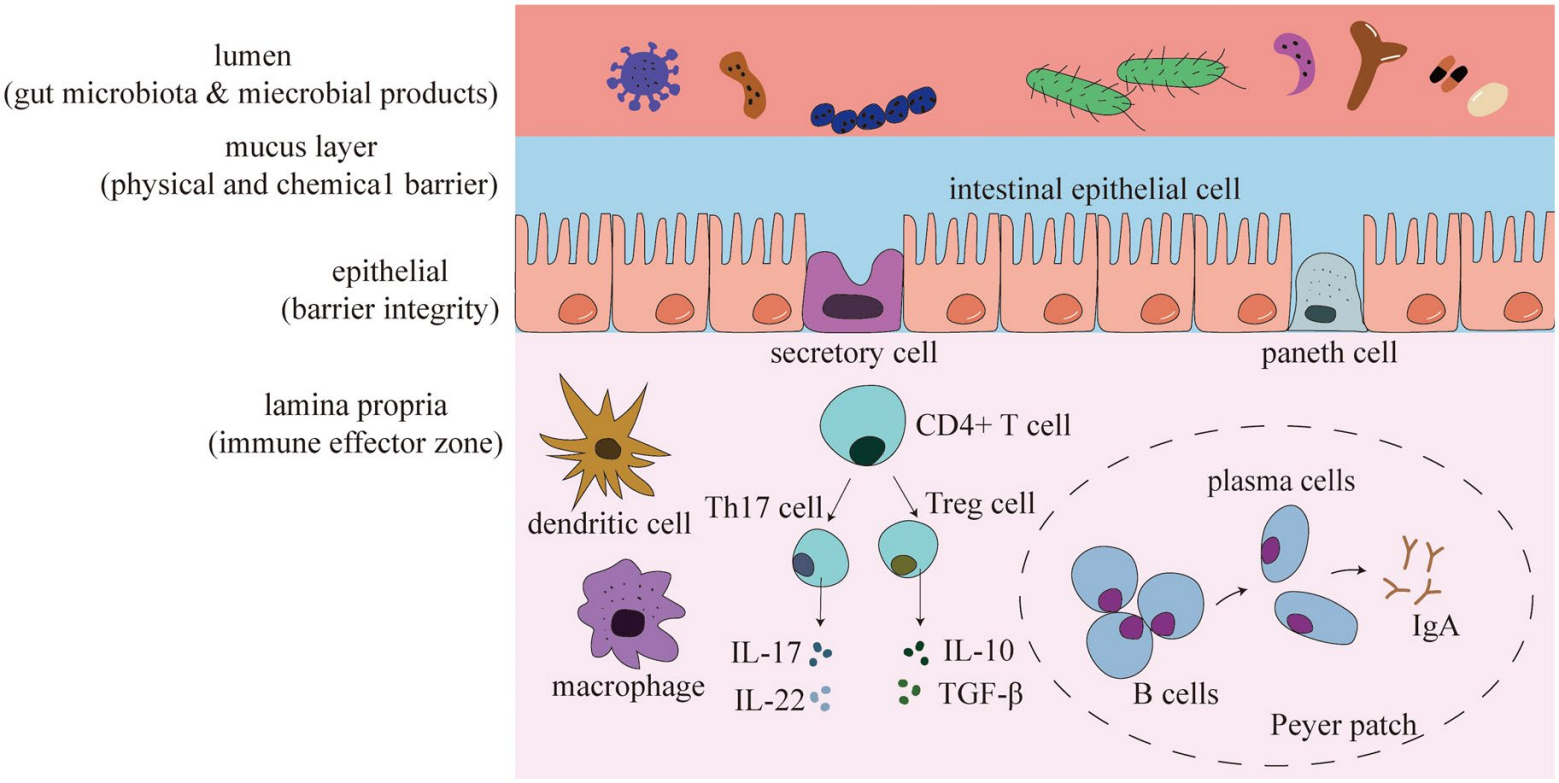
Composition and immune functions of the intestinal mucosal immune system. The intestinal mucosal immune system consists of the epithelial layer, lamina propria, and GALT. The epithelial layer forms the first line of defense by maintaining barrier integrity and mediating interactions between the gut microbiota and the host. The lamina propria contains diverse innate and adaptive immune cells and serves as a major immune effector site of the intestinal mucosa. GALT, including Peyer’s patches and isolated lymphoid follicles, functions as a key site for antigen sampling and the induction of gut-specific immune responses. Together, these components constitute the structural and functional basis of intestinal immune regulation, which links intestinal immunity to systemic inflammation and kidney-related outcomes within the gut-kidney axis.

The intestinal mucosal immune system is the largest immune barrier in the body and consists of epithelial cells, lamina propria, and gut-associated lymphoid tissue (GALT), which form a protective barrier to intestinal integrity [10] (Figure 1). Epithelial cells act as the first line of defense for intestinal immunity, avoiding aberrant immune responses by constructing physical and chemical barriers to isolate the intestinal microbiota from immune cells, thus establishing a host-symbiotic mutualistic relationship [11]. The lamina propria is located in the lower layer of the intestinal epithelium, which is the main immune effector zone of the intestinal mucosa and is rich in immune cells, including those from the innate and adaptive immune systems, such as dendritic cells(DC), macrophages, mast cells, B cells, and T cells, etc. There are important regional differences in the distribution of these immune cells along the length of the intestine [12]. Human GALT, including Peyer’s patches (PP), appendix and isolated lymphoid follicles (ILF), are key antigenic sampling and adaptive immune induction sites within the intestinal wall [13].

### Bidirectional regulatory mechanisms of the gut-kidney axis

**Figure 2.**
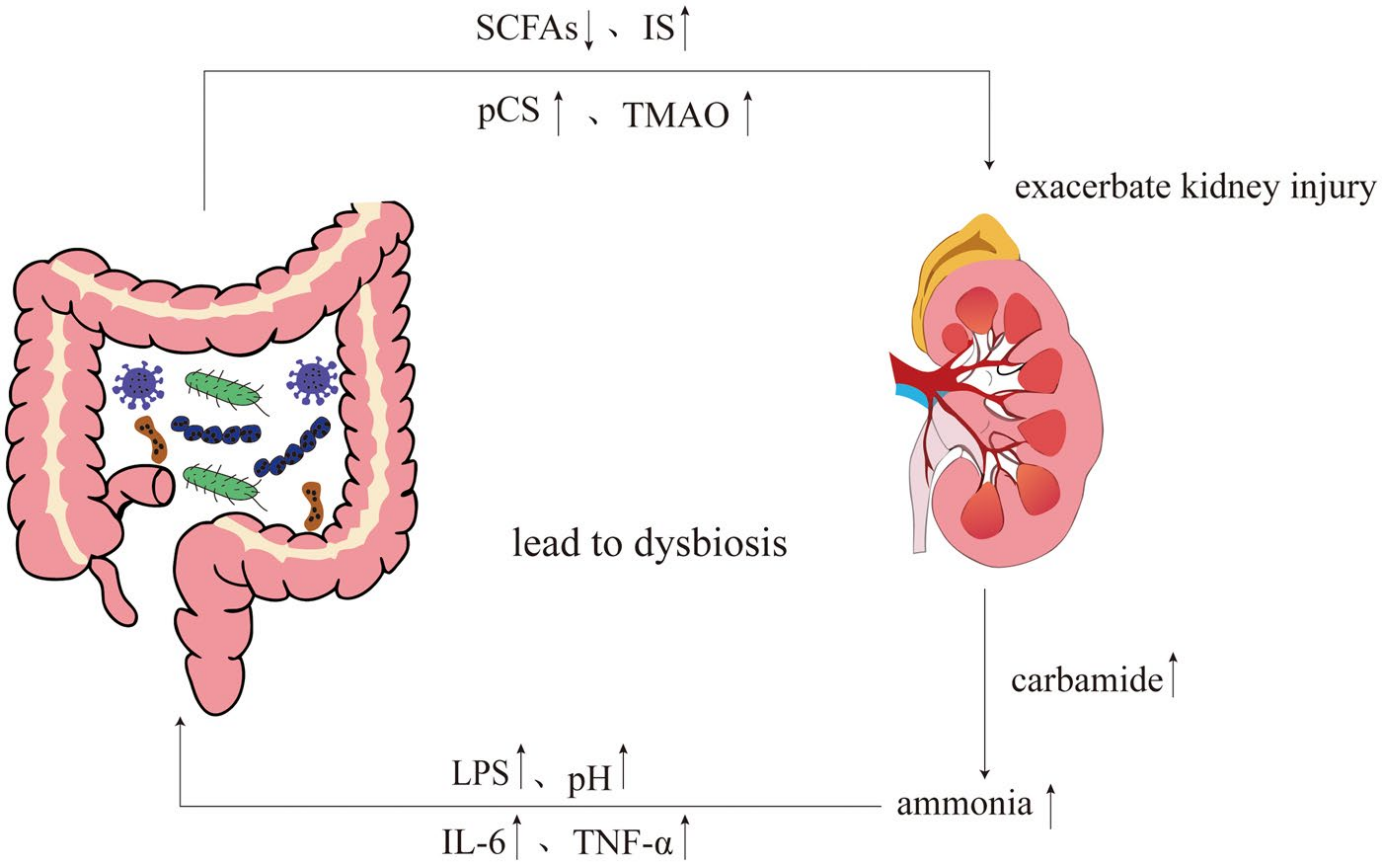
The bidirectional regulation of the gut-kidney axis. The gut-kidney axis achieves bidirectional regulation through metabolic, inflammatory, and immune pathways. Dysbiosis of the gut microbiota and intestinal barrier damage lead to the entry of bacterial metabolites, such as TMAO and indole sulfate, into the bloodstream, triggering systemic inflammation and exacerbating kidney damage. Conversely, impaired renal function leads to the accumulation of uremic toxins and urea retention, altering the intestinal microenvironment and pH, disrupting microbiota diversity, and impairing barrier function, thereby further exacerbating microbiota imbalance and intestinal inflammation.

The gut-kidney axis emphasizes the existence of complex and dynamic bidirectional regulatory mechanisms between the gut and the kidneys, and there is growing evidence that crosstalk between the host and the microbiota is bidirectional. On the one hand, the gut regulates renal function and immune status through microbial communities, metabolites, and immune factors; on the other hand, metabolic disorders and retention of urinary toxins in renal disease states in turn affect the gut ecology and immune barriers, forming a ‘closed-loop regulation’ [14] (Figure 2).

### Remote regulation of the kidneys by the gut

#### Metabolite-mediated effects

Gut-derived metabolites may be both beneficial to the kidney by promoting immune tolerance and pathological by inducing rejection and chronic inflammation (Table 1). Under healthy conditions, the gut microbiota produces various small-molecule metabolites by breaking down dietary fiber and proteins. Among these, short-chain fatty acids (SCFAs) play a crucial regulatory role between the host and the microbial community. SCFAs, primarily comprising acetate, propionate, and butyrate, exert key functions in modulating immune responses and maintaining intestinal barrier integrity [15]. As an energy source for colonic cells, SCFAs maintain intestinal homeostasis by regulating tight junction proteins [16]. Simultaneously, they modulate Treg cells’ differentiation by binding to G protein-coupled receptors (GPR41, GPR43, GPR109a) and inhibiting histone deacetylases (HDACs) [17], thereby suppressing inflammatory responses and safeguarding renal health. In addition to SCFAs, secondary bile acids represent another important class of gut microbiota-derived metabolites. Primary bile acids synthesized in the liver are converted into secondary bile acids in the intestine through the action of specific gut microbial communities. Beyond their classical roles in lipid and energy metabolism, secondary bile acids function as signaling molecules by activating the farnesoid X receptor (FXR) and the G protein-coupled bile acid receptor TGR5. Through these signaling pathways, secondary bile acids play critical roles in maintaining intestinal barrier integrity and regulating immune responses and inflammatory homeostasis [18]. In KTRs, gut microbiota dysbiosis may lead to alterations in bile acid metabolic profiles and associated signaling pathways, thereby influencing the immune microenvironment of the renal allograft and chronic inflammatory status *via* the gut-kidney axis [19]. During gut microbiota dysbiosis arising from various causes, the production of beneficial bacterial metabolites such as SCFAs diminishes. Concurrently, uremic toxins including indoxyl sulfate (IS), p-cresyl sulfate (pCS), and trimethylamine N-oxide (TMAO) gradually accumulate. This accumulation further induces oxidative stress, thereby promoting or exacerbating renal injury [20].

**Table 1.** Intestinal metabolites and their roles.

Intestinal metabolites	Major flora	Targeted pathways/cells	Functions	Reference
SCFAs (acetate, propionate and butyrate)	*Bacteroidetes、Firmicutes*	NF-κB、GPR41、GPR43 HSP25、HSP72 Treg cells	Anti-inflammatory, mediates metabolic processes and enhances intestinal barrier	[36]
P-Cresyl Sulfate	*Enterococcaceae, Clostridiaceae, Staphylococcaceae, Enterobacteriaceae*	leukocytes、RAAS/TGF-β pathway、ROS	Pro-inflammatory and cytotoxic	[37]
Indoxyl Sulfate	*Bacteroides* and *Clostridium*	ROS、vascular smooth muscle	Promotes oxidative stress and inflammation	[24]
TMAO	*Firmicutes* and *Proteobacteria*	PERK、ROS、NLRP3	Pro-inflammatory, promotes atherosclerosis and renal tubular injury	[38]

SCFAs: short-chain fatty acids; NF-κB: nuclear factor-kappa B; GPR41: G protein-coupled receptor 41; GPR43: G protein-coupled receptor 43; HSP: heat shock protein; ROS: reactive oxygen species; RAAS/TGF-β: renal renin angiotensin aldosterone system/transforming growth factor-beta; TMAO: trimethylamine N-oxide; PERK: protein kinase R-like endoplasmic reticulum kinase; NLRP3: domain-containing-3.

#### The intestinal barrier and inflammatory response

The normal intestinal barrier comprises a multi-layered structure that separates the internal environment from the external environment, serving as an antimicrobial barrier and maintaining the stability of the body’s internal environment [21]. In certain patients with renal disease, the gut microbiome homeostasis is disrupted. This leads to a reduction in tight junction proteins within the intestinal barrier, alterations in its structural integrity, and impaired barrier function. Consequently, increased intestinal permeability allows lipopolysaccharides (LPS), enteric bacteria, and harmful metabolic by-products to more readily enter the systemic circulation, a phenomenon termed ‘leaky gut’. This, in turn, triggers inflammatory responses within the kidneys and throughout the body [22]. LPS mediates increased intestinal tight junction permeability by enhancing Toll-like receptor 4 (TLR4) expression in intestinal epithelial cell membranes [23]. When these toxins enter systemic circulation, they activate immune cells to induce the release of inflammatory mediators such as IL-6 and TNF-α. Upon reaching the kidneys, they may also bind to renal cell TLRs, triggering or exacerbating inflammatory responses that lead to renal injury and fibrosis [24].

#### Neuroendocrine pathways

The gut microbiota may also indirectly regulate renal function by modulating neurotransmitter and hormone levels. Certain microbial strains synthesize acetylcholine (Ach) and gamma-aminobutyric acid (GABA), among other molecules, which indirectly influence renal haemodynamics by regulating sympathetic nervous system activity [25]. As an inhibitory neurotransmitter, GABA suppresses excessive sympathetic excitation, thereby reducing renal vasoconstriction and exerting indirect renal protective effects [26]. It has also been demonstrated that Ach promotes renal vasodilation, inducing a dose-dependent increase in renal blood flow, thereby playing a significant role in blood pressure regulation [27]. Gut microbiota dysbiosis can further activate the hypothalamic-pituitary-adrenal (HPA) axis, leading to elevated cortisol levels that mediate the progression of CKD [28].

### Reverse regulation of the gut by the kidneys

#### Effects of uremic toxins and metabolites

Normally, metabolic by-products such as uremic toxins are eliminated by the kidneys. However, when renal function declines, the levels of uremic toxins gradually accumulate within the body, creating a toxic environment in the bloodstream. This not only exerts adverse effects throughout the body but may also disrupt the intestinal environment [29]. For instance, persistent urea, under the action of urease-producing bacteria, generates ammonia. This not only alters the local pH and metabolic environment, leading to an imbalance in the microbial community, but also damages intestinal epithelial cells, causing increased permeability. Consequently, more toxins enter the circulation, further exacerbating renal deterioration [30]. Moreover, these toxins have been demonstrated to be associated with the development of cardiovascular diseases in individuals with CKD [31].

#### Inflammation and the immune response

Alterations in the intestinal barrier can modulate renal function *via* inflammatory signaling pathways. Conversely, renal function can influence the intestinal microenvironment through systemic inflammation and immune activation. In patients with CKD, renal impairment not only causes metabolic disorders but also induces systemic inflammation by producing pro-inflammatory cytokines. The release of numerous inflammatory mediators into the circulation can act upon the intestinal mucosa, altering the local microenvironment and leading to dysbiosis of the gut microbiota [5]. This harmful gut-kidney crosstalk has also been described in acute kidney injury (AKI). Reports indicate that in septic AKI, elevated levels of inflammatory cytokines such as IL-6, TNF-α, and IL-1β lead to intestinal wall ischemia, edema, and increased permeability. This further amplifies the systemic inflammatory response, resulting in various organ dysfunctions and even death [32].

#### Alterations in nutritional metabolism and intestinal motility

Patients with CKD exhibit structural and functional impairments in their intestinal epithelium. Beyond the aforementioned factors, this may also be associated with alterations in nutritional status and intestinal motility, such as reduced dietary fiber intake [33], extensive use of phosphate binders [34], and slowed intestinal transit time [35]. These factors may modify the gut’s nutrient supply and microenvironment, thereby selectively promoting the proliferation of certain harmful bacteria and further exacerbating dysbiosis.

### Influence of the gut microbiota on the outcome of kidney transplantation

The outcomes of kidney transplantation, encompassing graft survival, rejection reactions, and other associated complications, depend upon multiple factors. Recent research has revealed that the gut microbiota, serving as a crucial hub for the body’s immune and metabolic functions, plays a significant role in the prognosis of KTRs [39]. Influenced by various factors, the gut microbiome characteristics of KTRs differ markedly from those prior to transplantation (Table 2).

**Table 2.** Changes in flora after kidney transplantation.

Study population	Methods	Study design (RCT/Cohort/Animal)	Time after transplantation	Changes in the flora of KTRs	Reference	
26 KTRs	PCR and deep sequencing	Cohort	in the first 3 months after transplantation	*Proteobacteria* ↑		[44]
60 KTRs	PCR and sequencing	Cohort	at 1 and 6 months after transplantation	*Firmicutes*, *Bacteroidetes*, *Proteobacteria*, and *Actinobacteria*↑		[45]
19 KTRs	PCR and deep sequencing	Cohort	within the first month of transplantation	*Firmicutes*, *Actinobacteria*, and *Bacteroidetes*↑		[46]
20 KTRs	bioinformatics approach	Cohort	at least 6 months post-transplant	*Firmicutes* and *Bacteroidetes*↑		[47]
16 KTRs、84 CKD and 53 healthy subjects	deep sequencing	Cohort	–	*Firmicutes*, *Lachnospiraceae*, *Ruminococcaceae* and *Faecalibacterium* ↓ *Bacteroidetes*, *Proteobacteria*, *Clostridiales*, and *Enterobacteriaceae* ↑		[41]
139 KTRs、105 healthy subjects	16S rRNA sequencing	Cohort	at least one year post-transplantation	*Proteobacteria* ↑ *Actinobacteria*↓		[40]
40 KTRs and 18 healthy subjects	PCR and deep sequencing	Cohort	at least three months of transplantation	*Actinobacteria*, *Bacteroidetes*, and *Verrucomicrobia*↓ *Proteobacteria*↑		[48]
10 KTRs	deep sequencing and LC-MS methods	Cohort	on the 7th and 30th day after the operation	*Clostridiales*, *Clostridia*, *Ruminococcaceae*, *Faecalibacterium*, and *Veillonellaceae*↓ *Bacilli*, *Enterococcaceae*, and *Enterococcus*↑		[49]
12 KTRs and 12donors	metagenomic sequencing	Cohort	between four to eight weeks after transplantation	*Roseburia*, *Streptococcus*, *Oscillibacter* and *Romboutsia*↑		[50]
71 KTRs	16S rRNA gene V4-V5 deep sequencing	Cohort	in the first 3 months after transplantation	in diarrheal fecal specimens, *Ruminococcus*, *Dorea*, *Coprococcus*, and *Bacteroides*↓, *Enterococcus* and *Escherichia*↑		[51]
28 KTRs and 30 healthy controls	16S sequencing and metabolomics studies	Cohort	–	*Enterococcus*, *Escherichia*, *Shigella*, *Rothia*, and *Streptococcus*↑, *Faecalibacterium*, *Prevotella*, *Blautia*, *Ruminococcus*, *Agathobacter* and *Subdoligranulu*↓		[52]

Swarte et al. found that dysbiosis characterized by a general loss of microbial diversity was present in KTRs and that the use of mycophenolate mofetil and antibiotics was associated with changes in the gut microbiome in KTRs and was associated with lower diversity [40]. Altered flora structure is characterized by a decrease in functional flora and an increase in potentially pathogenic bacteria. For example, by comparing the intestinal flora of 16 KTRs with that of 84 CKD patients and 53 healthy subjects, one study found that the overall microbial structure of KTRs was similar to that of CKD patients and that both had significantly lower abundance of *Firmicutes*, *Lachnospiraceae*, *Ruminococcaceae* and *Faecalibacterium*, and significantly higher abundance of *Bacteroidetes*, *Proteobacteria*, *Clostridiales*, and *Enterobacteriaceae* were significantly more abundant [41].

**Figure 3.**
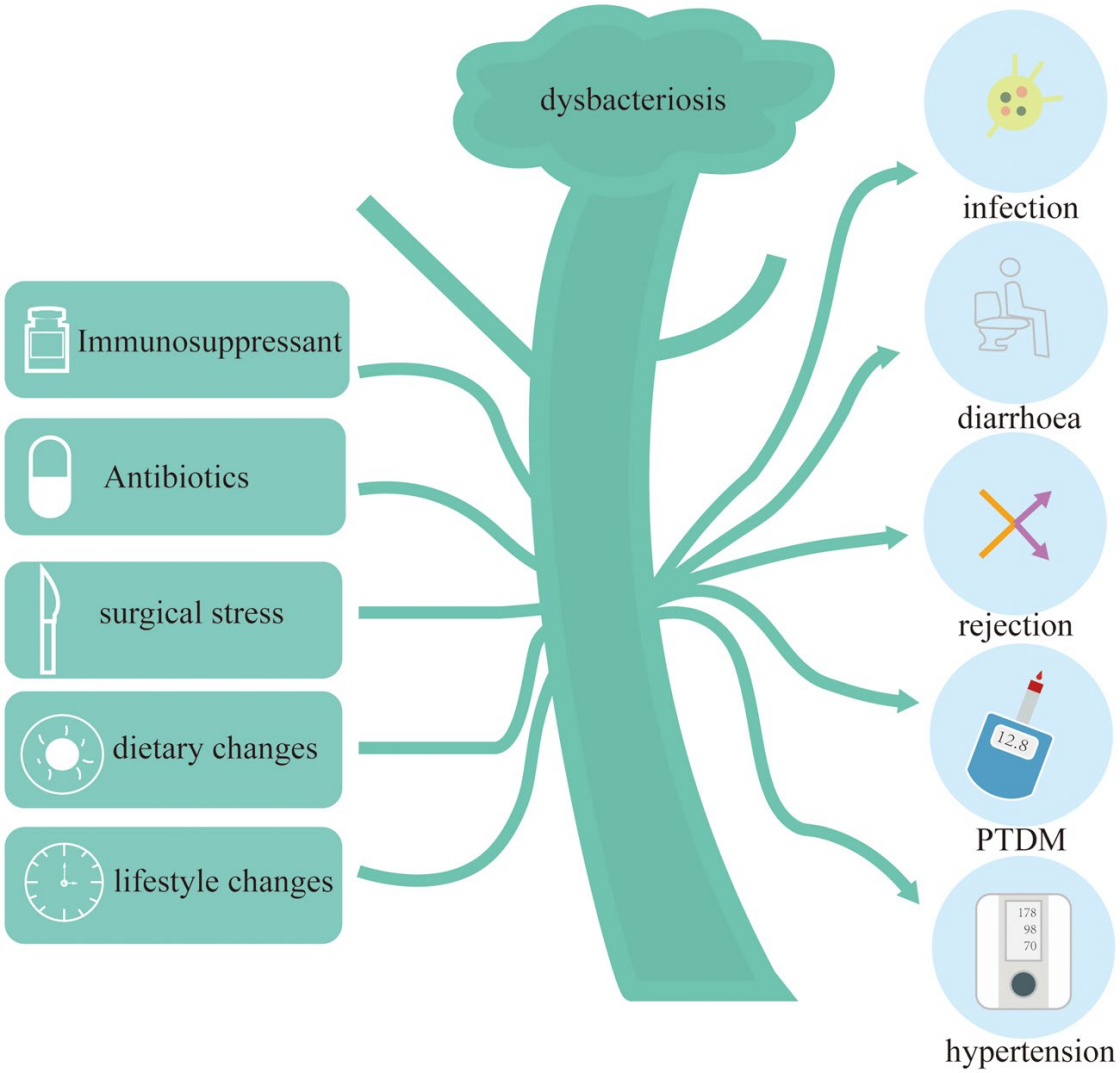
The causes and consequences of dysbiosis. Dysbiosis of the gut microbiota can be caused by various factors, including long-term use of immunosuppressants and antibiotics, surgical stress responses, changes in diet and lifestyle, and others. Dysbiosis leads to impaired gut barrier function, triggers systemic inflammatory responses, and is associated with immune rejection reactions, potentially exacerbating graft dysfunction. Additionally, dysbiosis can cause nutritional metabolic abnormalities, increased susceptibility to infection, and elevated risk of cardiovascular events, significantly impacting the long-term prognosis of kidney transplant recipients.

Probiotics have been demonstrated to confer multiple benefits in solid organ transplantation, including anti-inflammatory effects, immune system enhancement, and maintenance of immune homeostasis [42]. However, KTRs frequently experience gut microbiota dysbiosis postoperatively due to multiple factors such as immunosuppression and infection. This dysbiosis is closely associated with transplant-related complications (Figure 3) and drug metabolism [39,43].

### Dysbiosis and post-transplant complications

#### Infections

The spectrum of intestinal flora changes after kidney transplantation, and new dominant flora may become a source of infection, which is a major cause of morbidity and mortality in KTRs [53]. Urinary tract infection (UTI) is a common infectious complication of KTRs, and a number of studies support a role for the gut microbiota in the pathogenesis of UTI. A study of 168 KTRs proposed that the abundance of *Escherichia intestinalis* was associated with the occurrence of *Escherichia* bacteriuria and UTI, and found that increased abundance of *Faecalibacterium* and *Romboutsia* was associated with a reduced risk of *Enterobacteriaceae* bacteriuria and UTI in KTRs, contributing to the development of intestinal microbial-based therapies for the prevention of recurrent UTI in KTRs [54].

#### Diarrhea

Diarrhea is a common complication after kidney transplantation, with a cumulative incidence of approximately 22% within three years post-transplant, according to a historical cohort study. Most post-transplant diarrhea is noninfectious diarrhea, i.e., no clear pathogen is identified, and is often associated with an immunosuppressive regimen containing tacrolimus and mycophenolate mofetil [55]. Recently, it has also been noted that diarrhea after kidney transplantation is also closely related to intestinal flora dysbiosis. For example, in a prospective study conducted by Lee et al. by collecting pre-transplantation and post-transplantation fecal specimens from 26 KTRs, it was found that patients with post-transplantation diarrhea had a significant reduction in *Bacteroides*, *Ruminococcus*, *Coprococcus*, and *Dorea*, and it was hypothesized that the reduction of commensal flora in the gut caused a metabolic disordered state and thus led to the diarrhea [44].

#### Rejection

Rejection, as one of the most serious immune complications after renal transplantation, especially T cell-mediated rejection (TCMR) and antibody-mediated rejection (AMR) is a key factor affecting graft survival [56]. A growing body of evidence suggests a correlation between the gut microbiota and the immune system. Animal studies have demonstrated that diet can induce transplantation tolerance by altering gut microbiota composition [57]. A high-fiber(HF) diet is thought to prevent ecological dysregulation of allograft receptors. Wu et al. found that HF diet-fed mice survived longer and had a reduced probability of acute and chronic allograft rejection compared with mice fed a normal diet. A significant increase in the relative abundance of *Bifidobacterium*, *Clostridium*, and *Bacteroidetes spp*. was HF diet-fed mice, which modulated tissue inflammation through the production of SCFAs acting on a variety of cell types, such as Treg cells, DCs and macrophages. This promotes allogeneic kidney graft survival and limits transplant-induced ecological dysregulation [58]. In a study of 35 KTRs divided into rejection, dysfunction and control groups, combined gut microbiome and metabolome analyses found that the rejection group was enriched in *Escherichia* and *Faecalibacterium* [59], which have been shown to elicit increased systemic IgG responses in the presence of intestinal barrier disruptions, potentially leading to immune activation in the host [60]. These findings highlight that an imbalance in the gut microbiota can alter renal function and that affect metabolic pathways and metabolite abundance. Targeting gut microbiota composition may therefore be a promising strategy to improve graft tolerance. Overall, careful monitoring of the gut microbiota and consideration of dietary interventions may help enhance graft survival in kidney transplant recipients.

### Posttransplantation diabetes mellitus (PTDM)

PTDM is a common metabolic complication after kidney transplantation, affecting approximately 10 ∼ 45% of KTRs, and is associated with a significantly increased risk of major adverse cardiovascular events and cardiovascular death [61]. To a large extent, PTDM is thought to be associated with immunosuppressive regimens [62]. However, emerging evidence also suggests that gut dysbiosis-associated functional alterations may be involved in the pathogenesis of PTDM, although a direct causal relationship independent of immunosuppressive effects has not been established [63]. Recent observational studies have reported an increased relative abundance of *Lactobacillus sp*. and a marked reduction in *Akkermansia muciniphila* in patients with PTDM, suggesting that these microbial alterations may be associated with an increased susceptibility to PTDM rather than serving as independent risk factors [64]. Furthermore, a subsequent comprehensive analysis of the gut microbiota in PTDM patients indicated that reduced SCFAs biosynthesis capacity, likely reflecting a decreased abundance of SCFA-producing bacteria, was associated with PTDM development [65].

#### Hypertension

Dysbiosis of the gut flora may also promote another common complication after kidney transplantation, namely hypertension. This is often associated with the use of immunosuppressive agents such as tacrolimus which may lead to vascular endothelial dysfunction by decreasing endothelial nitric oxide synthase (eNOS) expression or activity, resulting in hypertension. Further studies have found that *Lactobacillus fermentum* CECT5716 (LC40) ameliorates tacrolimus-induced hypertension [66].

A few common post-operative complications following kidney transplantation and associated changes in the microbiota are presented in Table 3.

**Table 3.** Intestinal flora and complications after kidney transplantation.

Complication	Microbiota changes	Possible mechanisms	Key evidence	Clinical implication	Reference
Diarrhea	*Bacteroides*, *Ruminococcus*, *Coprococcus*, and *Dorea*↓	a commensal role in competing out pathological microbiota and an inability to effectively digest carbohydrates	Cohort studies	highlight a potential association between the gut microbiota and infectious complications following kidney transplantation.	[44]
Rejection	*Bacilli* and *Lactobacillales*↑, *Clostridia*, *Clostridiales* and *Faecalibacterium*↓	the gut microbiota may affect AMR through regulating the metabolic pathways	Cohort studies	provide a foundation on the role of gut microbiota in AMR after kidney transplantation, and potentially support novel diagnostic biomarkers and therapeutic options for AMR.	[67]
PTDM	*Lactobacillus sp*.↑, *Akkermansia muciniphila*↓	immunosuppressive treatment and other factors might aggravate a ‘predysbiotic microbiota’, contributing to the metabolic disorders	Cohort studies	providing the best treatment, lifestyle coaching and medical follow-up for KTRs.	[64]
Urinary tract infection	*Faecalibacterium* and *Romboutsia*↓	high relative abundances of *Faecalibacterium* and *Romboutsia* are associated with decreased risk for *Enterobacteriaceae* bacteriuria and UTI	Cohort studies	support future studies evaluating the use of gut microbial based therapies for the prevention of recurrent UTIs in KTRs.	[54]
Hypertension	*Firmicutes* and *Bacteroidetes*↑	gut dysbiosis induced by tacrolimus contributes to hypertension and endothelial dysfunction	Animal experiments	open new possibilities to increase the benefit-risk ratio of immunosuppressor drug by modulation of microbiota with safe probiotic bacteria.	[66]

### Gut microbiota and drug metabolism

Immunosuppressants, as one of the key therapeutic approaches for KTRs, have been shown to interact with the host’s gut microbiota. On the one hand, immunosuppressant administration may alter the composition and function of the gut microbiota; on the other hand, the gut microbiota may directly or indirectly influence the pharmacokinetic characteristics of immunosuppressants [4]. Mycophenolate mofetil (MMF) is hydrolyzed *in vivo* to mycophenolic acid (MPA), with its metabolite MPA glucuronide (MPAG) ultimately excreted *via* bile and feces. Certain gut microbiota, such as *Bacteroides* species, secrete β-glucuronosidase enzymes that hydrolyze MPAG back to MPA, thereby influencing drug absorption [68]. The therapeutic window for the immunosuppressive agent tacrolimus is narrow, with its oral bioavailability being low and highly variable. Multiple factors have been implicated in this phenomenon, and recent studies have highlighted the role of the gut microbiota [69]. Lee et al. employed 16S rRNA deep sequencing to analyze the fecal microbiota of 19 KTRs in the early postoperative period. They discovered a positive correlation between *Faecalibacterium prausnitzii* abundance and tacrolimus dosage [46]. Subsequently, building upon this foundation, Guo et al. further demonstrated through experimentation that certain commensal gut bacteria can metabolize tacrolimus into a novel metabolite, M1 (9-hydroxy tacrolimus), whose pharmacological activity may be lower than that of tacrolimus [43]. This finding was subsequently validated in 10 KTRs [70]. This evidence indicates that the gut microbiota is a significant factor mediating inter-individual pharmacokinetic variability in KTRs. It holds promise as a key entry point for optimizing immunosuppressant dose management and achieving personalized treatment in the future.

### The intestinal immune system and the immune microenvironment after kidney transplantation

Intestinal immune cells act as critical downstream effectors of gut microbiota-derived signals and play a pivotal role in shaping immune tolerance and rejection after kidney transplantation. While microbial metabolites such as SCFAs were introduced earlier in the context of gut-kidney remote regulation, there is growing evidence indicates that these metabolites also directly shape intestinal immune cell composition. In particular, SCFAs promote Treg cells’ differentiation while suppressing Th17 cell responses, and modulate macrophage polarization toward an anti-inflammatory M2 phenotype, thereby contributing to immune tolerance after kidney transplantation. In this section, we focus on key intestinal immune cell populations and discuss how microbiota-driven immune modulation contributes to immune homeostasis or rejection following renal transplantation, highlighting potential targets for therapeutic intervention.

### Regulatory T (Treg) cells

Treg cells are a specialized subset of CD4 T cells with immunosuppressive functions, typically characterized by the expression of Foxp3, a master transcription factor that is essential for Treg development and suppressive function [71]. Activated Treg cells play an important role in maintaining autoimmune tolerance and preventing over-immune responses through the production of cytokines such as IL-10, TGF-β and IL-35 [72]. The intestinal environment, especially specific commensal flora and their metabolites such as SCFAs may enhance tolerance to rejection during kidney transplantation *via* Treg cells and GPR43 receptors in renal tissue [58].

Furthermore, it has been noted that donor-derived Treg cells have been shown to produce more cytokines such as IL-10, IFN-γ and TGF-β, which help maintain graft function and tolerance [73]. Therefore, promoting intestinal Treg cells expansion by maintaining intestinal microecological stability may be an important strategy for inducing immune tolerance to transplantation. This has been demonstrated in animal models, and several clinical trials targeting Treg cell therapies and kidney transplantation related therapies are already underway [74].

### Th17 cells

T lymphocytes with the surface marker CD4 can differentiate into two subpopulations of cells with opposite immune functions, a Treg subpopulation and a pro-inflammatory subpopulation of Th17 cells. Therefore, maintaining the balance of Th17/Treg cells plays a key regulatory role in maintaining intestinal and parenteral immune homeostasis [75]. Th17 cells are important effector cells involved in inflammation and rejection, activating inflammatory pathways by secreting cytokines such as IL-17 and IL-22, which in turn participate in various inflammatory responses [76]. The relationship between Th17 cells and the development of inflammation in a variety of renal diseases, such as crescentic glomerulonephritis, lupus nephritis, and IgA nephropathy, has been elucidated in detail [77]. The finding of increased local expression of IL-17 in graft rejection suggests that Th17 may be an important player in causing acute rejection or chronic allograft dysfunction [78]. Treg/Th17 imbalance is thought to be associated with a variety of diseases, including post-transplant rejection, so studies have attempted to modulate the Treg/Th17 balance through SCFAs, probiotics or specific medications to attenuate the immune response to transplantation, with initial efficacy demonstrated [79].

### Natural killer (NK) cells

NK cells are an important part of the innate immune system and, as natural cytotoxic cells, have the ability to act independently of antigen presentation [80]. In addition to their role in antiviral infection and antitumour defence, NK cells are involved in regulating the immune response in renal allograft recipients [81]. NK-cell activity is tightly regulated through a process known as education, which depends on interactions between inhibitory killer-cell immunoglobulin-like receptors (KIRs) and self-MHC class I (MHC-I) molecules, thereby shaping NK-cell responsiveness in the transplant setting [82]. Moreover, NK cells contribute to antibody-mediated rejection *via* CD16-dependent antibody-dependent cellular cytotoxicity (ADCC), linking donor-specific antibodies (DSA) to endothelial injury and microvascular inflammation [83].

On the one hand, NK cells are involved in acute and chronic allograft rejection; On the other hand, specific NK-cell subsets and functional states may also promote allograft tolerance through the secretion of immunoregulatory cytokines such as IL-10 [84,85].

### Dendritic cells (DCs)

DCs are important antigen-presenting cells (APCs) in the mucosal immune system and play important roles in innate and adaptive immunity, self-tolerance, and tissue repair [86]. Classical DCs are divided into two main subpopulations, CD8α/CD103 (cDC type 2) and CD11b (cDC type 1), which perform different immune functions. CD8α (present in lymphoid tissues) and CD103 (present in non-lymphoid tissues) play a major role in the delivery of antigens to CD8 T cells *via* MHC-I, while the CD11b subpopulation is better at inducing CD4+ T cell proliferation [87]. Ischemia/reperfusion injury (I/R) in renal grafts is a major cause of late graft loss, and studies have pointed to the ability of renal DCs to participate in the inflammatory response to IR through the production of pro-inflammatory cytokines and other soluble inflammatory mediators, either directly or through the activation of effector T-lymphocytes and natural killer T-cells (NKT) [88].

### Macrophages

Macrophages are multifunctional cells of the innate immune system with multiple functions such as antigen presentation, phagocytosis of pathogens, regulation of inflammation and tissue repair. Based on their phenotype and function, macrophages can be classified into M1 type with pro-inflammatory function and M2 type with anti-inflammatory function [89]. A significant decrease in M2 macrophages and a significant increase in M1 macrophages were found in renal transplant recipients who developed graft dysfunction or rejection [90]. The polarization state of macrophages can be significantly modulated by gut microbiota-derived metabolites, such as SCFAs, tryptophan (Trp) and bile acids (BA) metabolites can promote differentiation from M1 to M2 macrophages [91]. Thus, by regulating the direction of intestinal macrophage polarization, it may provide a novel target for immune intervention after renal transplantation.

### Potential intervention strategies targeting the gut-kidney axis

**Figure 4.**
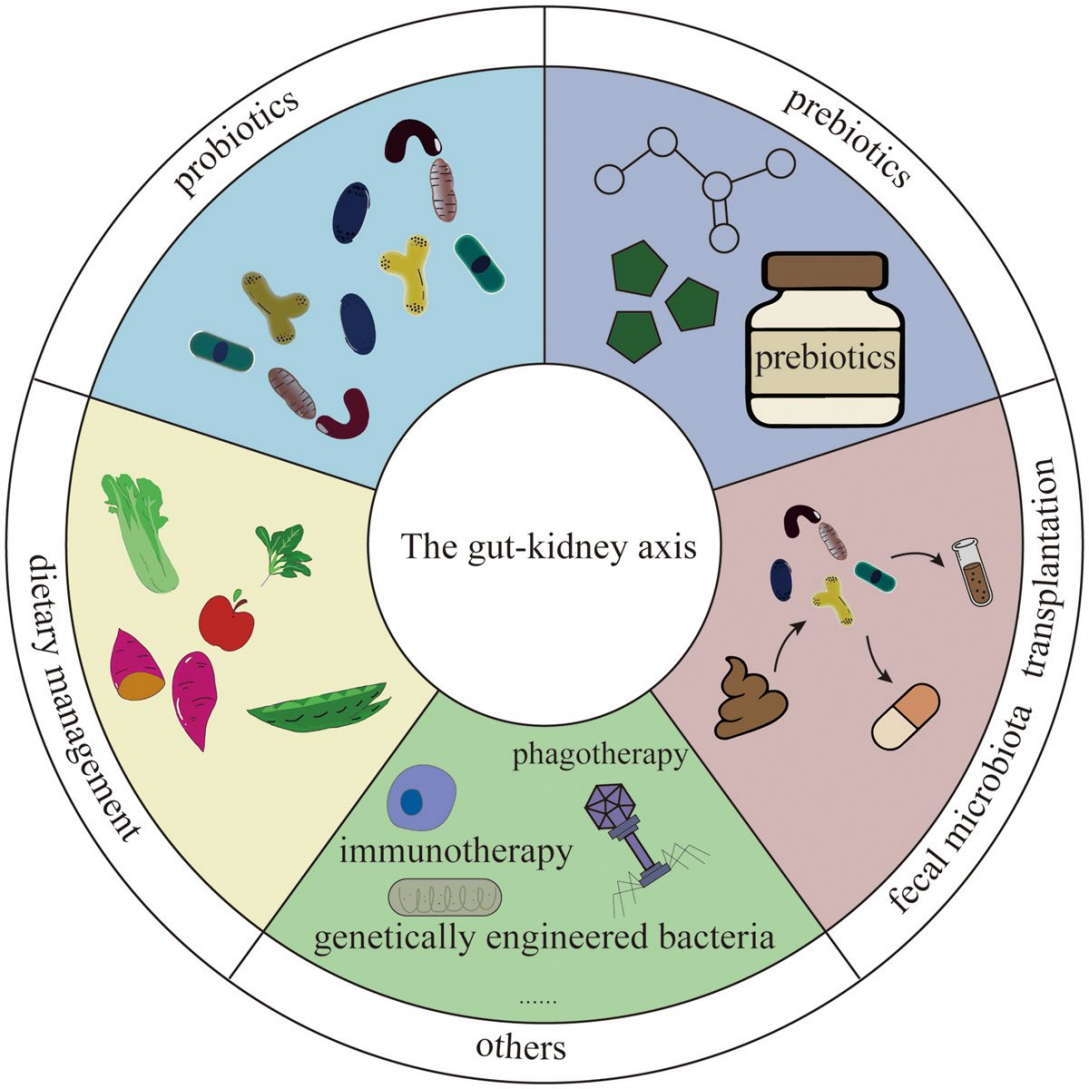
Intervention strategies targeting the gut-kidney axis. Improving the prognosis of KTRs by regulating the intestinal microbiota. Intervention measures include the use of probiotics and prebiotics, which can reshape the microbiota structure, enhance barrier function, and reduce inflammatory responses; dietary interventions such as high-fiber, low-protein diets help promote the growth of beneficial bacteria and improve the metabolic environment; FMT, as a new strategy for rebuilding the microbiota, shows potential in certain refractory microbiota disorders; Other interventions include immunotherapy, bacteriophage therapy, and genetically engineered bacteria.

Potential intervention strategies targeting the gut-kidney axis have attracted increasing interest as adjunctive approaches to improve outcomes after kidney transplantation. These strategies aim to restore microbial homeostasis, modulate intestinal immunity, and regulate microbe-derived metabolites. Figure 4 provides an overview of the major categories of gut-kidney axis-targeted interventions currently under investigation, including dietary management, microbial therapies, and other emerging approaches. Given the heterogeneous nature and variable quality of the available evidence, we further provide a structured summary of the evidence supporting each intervention, including study design, key findings, and major limitations (Table 4), to facilitate critical appraisal and clinical interpretation.

**Table 4.** Levels of evidence for gut-kidney axis-targeted interventions in KTRs.

Intervention	Evidence type	Key findings	Limitations	References
Dietary management	Animal experiments；narrative review and observational human data；observational studies	dietary supplementation with high-fiber promotes renal allograft survival and limits transplant-induced dysbiosis；the Mediterranean and the DASH diets are the most beneficial dietary patterns in KTRs	Well-designed RCTs are lacking; the direct impact of specific dietary interventions on graft rejection and long-term outcomes remains uncertain	[92]
Probiotics	Small RCT; animal experiments; observational studies	*Lactobacillus* mixture intervention could improve kidney function; a higher incidence of decreasing creatinine in KTRs using *Lactobacillus* mixture	Limited sample sizes；short intervention duration；lack of data on long-term graft outcomes	[93–95]
Prebiotics	Small RCT	prebiotics may reduce infections and gastrointestinal symptoms in KTRs	Small sample sizes; short study duration	[96,97]
FMT	Case reports; observational studies	treats rCDI；contributes to the drastic reduction in fecal VRE abundance；alleviates immunosuppressant-associated diarrhea and recurrent UTI in KTRs	Safety concerns；limited data；needs long-term observation；no RCTs in KTR	[98–100]
Other therapies	Conceptual	modulate gut microbial burden, selectively eliminate pathogenic taxa, neutralize gut-derived toxins, or restore immune homeostasis through targeted or adjunctive strategies	No data in KTRs；health benefits and safety is insufficient	[101]

### Dietary management

Diet, as one of the most direct and controllable factors influencing the gut microbiome, is an important direction in regulating the function of the gut-kidney axis [102]. Diet can provide substrates for the metabolism of gut microbes, which directly or indirectly contribute to gut health and influence systemic inflammation, immunity and metabolism through the production of certain metabolic by-products [61]. For example, dietary carbohydrates in the diet can be metabolized by certain specific intestinal flora to produce SCFAs, including acetate, propionate and butyrate, which on the one hand enhance the stability of the intestinal epithelial barrier, and on the other hand promote the formation of an anti-inflammatory immune microenvironment in the intestine [36]. Dietary modifications therefore have a moderating effect on the health of KTRs, who are advised to adopt the Mediterranean and DASH (Dietary Approaches to Stop Hypertension) diets, which are high in fiber and rich in fresh plant foods, and dietary patterns that place greater emphasis on increasing intake of fresh plant foods and decreasing intake of processed foods and meat [103]. Diet is not only a basic need for maintaining nutritional status, but also an important factor in regulating intestinal microecology and influencing immune response and metabolic balance. Therefore, dietary intervention strategies based on the gut-kidney axis are becoming an economical, safe and actionable adjunctive therapy.

### Microbial therapies

KTRs are in a state of long-term immunosuppression, metabolic disorders and impaired intestinal barrier function, and the intestinal flora is highly susceptible to imbalance, which triggers a series of post-transplantation complications. Therefore, microbial therapies based on the improvement of the imbalance of the bacterial flora, the protection of the intestinal barrier, and enhancement of the function of the immune system are being widely used in the transplantation population [9]. Microbial therapies, which include several forms of intervention, mainly probiotic and prebiotic supplements and fecal microbial transplantation (FMT).

### Probiotics

Probiotics are live microorganisms that, when ingested in sufficient quantities, provide health benefits to the host, and include mainly Bifidobacterium spp, Lactobacillus spp and Streptococcus spp [104]. Studies have shown that probiotics communicate with the host by regulating key signaling pathways (e.g., NF-κB and MAPK) and have a variety of functions such as regulating the barrier function of intestinal epithelial cells, enhancing mucosal immunity, regulating cytokine secretion, influencing T-lymphocyte populations and enhancing antibody secretion [105]. The administration of *Lactobacillus* preparations to KTRs not only prevents and treats diarrhea, but also reduces the incidence of *Clostridium difficile* infection [106]. The combination of *Lactobacillus plantarum* and *Lactobacillus paracasei* (Lm) has also been shown to have a high correlation with the incidence of creatinine reduction in KTRs, providing a potential strategy for reducing the dose of immunosuppressive drugs [95].

### Prebiotics

Prebiotics are non-digestible food components that selectively promote the growth of beneficial intestinal bacteria [107], and their main mechanism of action is to increase the production of SCFAs and lower the pH of the intestinal tract [4]. Postoperative supplementation of KTRs with probiotic preparations not only promotes the growth of probiotics such as *Bifidobacterium* and *Lactobacillus* in the body, but also reduces the level of urinary toxins and promotes the secretion of downstream glucagon-like peptide 1, which is an amelioration of the metabolic disorders that are common in KTRs [9]. In a study looking at 27 KTRs with gastrointestinal symptoms, it was found that they significantly relieved their gastrointestinal symptoms after receiving probiotics for 7 weeks [96]. It has also been shown that probiotic supplementation significantly improves gut microbial abundance and diversity in KTRs [97]. Currently, most of the applications of probiotic therapies have been used in CKD populations, and their use in KTRs needs to be further explored.

### Fecal microbiota transplantation (FMT)

FMT is a therapeutic method of transplanting functional gut flora from the feces of a healthy donor into a recipient either orally or by instillation to reestablish his or her intestinal microecology, and was initially used for the treatment of *recurrent C. difficile* infection (rCDI) [9]. Research on FMT in KTRs is only in its preliminary stages, focusing on case reports with small samples in exploratory studies. One case reported the successful use of FMT for rCDI in a cardiac renal transplant recipient who had no further CDI or *vancomycin-resistant Enterococcus* (VRE) infections and did not require hospitalization at 1-year follow-up, suggesting a better clinical outcome [99]. Subsequently, there have also been reports related to FMT for the treatment of KTRs with recurrent urinary tract infections (rUTI) [108]. Although FMT has been used in the treatment of inflammatory bowel disease, metabolic syndrome, autoimmune diseases and post-transplant infections and diarrhea [109], current evidence in KTRs remains limited by small sample sizes, short follow-up periods, and heterogeneous study designs. Importantly, serious donor-derived infections, including bacteremia caused by multidrug-resistant organisms, have been reported [110], underscoring the need for rigorous donor screening and caution before routine clinical application in immunosuppressed recipients.

### Other therapies

Beyond traditional dietary and microbiome remodeling approaches, several supplementary or novel interventions exist. These include selective digestive tract decontamination (SDD), which combines anti-infective and gastrointestinal cleansing effects by locally applying antibiotics to suppress inflammation and restore the intestinal barrier [111]. Interventions targeting bacterial metabolites also exist, encompassing both supplements that target beneficial metabolites [111] and oral adsorbents like AST-120 designed to eliminate harmful metabolites [112]. Other more targeted and precise microbiome intervention approaches exist, such as phage therapy [101], immunotherapy [113], and genetically engineered bacterial therapies [25]. However, these treatments have only been reported for use in CKD populations or remain at the animal experimentation stage, with limited evidence in KTRs. Their clinical value requires further validation through additional trials.

### Prospects and conclusions

In recent years, the ‘gut-kidney axis’, as an emerging theory of bidirectional regulation between intestinal microecosystems and kidneys, has been gaining widespread attention in the field of renal diseases, especially in the management of post-transplantation kidney disease. Studies have shown that the gut is not only an important organ for nutrient absorption and barrier defence, but also an important regulatory center that influences host immune homeostasis and graft fate. Long-term use of immunosuppressants and broad-spectrum antibiotics in KTRs will easily lead to a decline in the diversity of intestinal flora, barrier dysfunction and imbalance of immune regulation, which will induce a series of complications, such as postoperative diarrhea, infections, rejection and post-transplantation diabetes mellitus. Therefore, targeted regulation of the intestinal-renal axis and reestablishment of the balance of flora-barrier-immunity has become an important direction to improve the success rate of transplantation and the quality of life of patients. Importantly, this concept provides a unifying framework linking intestinal immunity, microbial metabolism, and allograft outcomes, thereby opening new avenues for immunomodulatory strategies beyond conventional pharmacological approaches.

Initial progress has been made in the exploration of the gut-kidney axis, from mechanistic studies to clinical interventions. A large number of basic studies have revealed the complex interrelationships between the gut microbiota, immune cells and the renal immune response. Gut microbes regulate intestinal immunity, endothelial function, and systemic inflammation levels by producing metabolites such as SCFAs, secondary bile acids, and urotoxins. In addition, specific gut flora play key roles in maintaining immune tolerance, suppressing renal inflammation and promoting renal function protection. In clinical studies, probiotics, prebiotics, and FMT have achieved some efficacy in kidney transplant recipients. Probiotics help regulate the immune response and reduce postoperative complications by restoring gut microbial diversity, increasing the production of SCFAs, and improving gut barrier function. FMT, as a relatively novel therapeutic modality, has also shown potential in improving the structure of intestinal flora and reducing immune rejection. However, the existing evidence remains heterogeneous, and the translational relevance of these findings varies substantially across experimental models and clinical settings.

Although the ‘gut-kidney axis’ intervention strategy has shown significant clinical potential, several challenges remain: individual differences in flora. Gut flora varies significantly among KTRs, and a single probiotic or prebiotics may not be appropriate for all patients. Further research is still needed on how to target specific flora imbalances through personalized microecological intervention programmes; The paradox of immunosuppression and flora imbalance. KTRs are usually in a state of long-term immunosuppression, which may have an inhibitory effect on the activity of probiotics, or may make some microorganisms that should be commensal bacteria become pathogenic. How to balance immunosuppression and flora reconstruction is the direction that current research needs to work on; Insufficient clinical evidence. Most of the studies are still at the stage of basic research or small-scale clinical trials, and there is a lack of sufficient large-sample, multicenter randomized controlled studies. More clinical data support, especially long-term follow-up studies, are needed in the future to validate the safety and efficacy of gut-kidney axis interventions. Specific standards for the use of probiotics, prebiotics, FMT and other treatments, dosages, and safety assessments remain imperfect. How to standardize treatment protocols, reduce potential side effects, and ensure individualization and precision of treatment is a key focus for future research. To address these challenges, future studies should integrate multi-omics approaches, including metagenomics, metabolomics and immune profiling, to identify predictive biomarkers that enable patient stratification and personalized intervention strategies. In addition, carefully designed multicenter randomized controlled trials with clinically meaningful endpoints are urgently needed to validate both efficacy and long-term safety.

Therefore, the focus of future research may be toward further in-depth exploration of precise microecological interventions, combined interventions by multiple means, and novel drug development. From a clinical translation perspective, dietary modification, particularly fiber-enriched diets, represents the most immediately actionable and low-risk intervention, whereas microbiota-based therapies such as probiotics and FMT should be applied cautiously within standardized protocols. More advanced strategies, including engineered microbial therapies or targeted manipulation of microbial metabolites, remain promising but are likely to represent long-term goals rather than near-term clinical solutions. Importantly, enthusiasm generated by compelling preclinical data must be balanced against the complexity of the human microbiome and the vulnerability of immunosuppressed transplant recipients.

The study of the gut-kidney axis provides a new way of thinking about immunomodulation and graft protection after kidney transplantation. By regulating the gut microbiota, immune cells and their metabolites, it is expected to provide more precise and personalized therapeutic solutions for KTRs in the future. Although the current study is still in the preliminary stage, the application of the gut-kidney axis intervention strategy in kidney transplantation is promising as basic research and clinical trials continue to deepen. Future studies will further advance this field and provide more effective treatments for KTRs to improve their long-term prognosis. Ultimately, continued integration of mechanistic insights with rigorously designed clinical studies will be essential to translate the gut-kidney axis from concept to clinically actionable precision immunomodulation.

## Data Availability

Data sharing is not applicable to this article as no new data were created or analyzed in this study. As a review article, all discussed information and conclusions are based on previously published studies, which are cited in the reference list.
